# Thank you to Virology Journal’s peer reviewers in 2014

**DOI:** 10.1186/s12985-015-0253-0

**Published:** 2015-04-18

**Authors:** Linfa Wang

**Affiliations:** CSIRO Animal Food and Health Sciences, Australian Animal Health Laboratory, Geelong, VIC 3220 Australia; Program in Emerging Infectious Diseases, Duke-NUS Graduate Medical School, Singapore, 169857 Singapore

## Abstract

The editors of *Virology Journal* would like to thank all our reviewers who have contributed to the journal in Volume 11 (2014). The success of any scientific journal depends on an effective and strict peer review process and *Virology Journal* could not operate without your contribution. We are grateful to the large number of reviewers, who have done a great job in not only lifting the quality of the journal’s scientific peer reviewing process, but also helped us to reach a median time to first decision of 39 days.

We look forward to your continuous support of *Virology Journal* either as an invited reviewer or a contributing author in the years to come.

**Maria Aamelfot**

Norway

**Jorge Abad**

USA

**Ahmed Abd El Wahed**

Germany

**Jônatas Abrahão**

Brazil

**Akio Adachi**

Japan

**Claudio Afonso**

USA

**Stephen Ahern**

USA

**Jameleddine Aissa Larousse**

Tunisia

**Mohammed Al-Ahdal**

Saudi Arabia

**Annika Allard**

Sweden

**Andrew Allison**

USA

**Sidney Altman**

USA

**Barry Alto**

USA

**Alberto A Amarilla**

Brazil

**Jaime Henrique Amorim**

Brazil

**Maria Joao Amorim**

Portugal

**Nisalak Ananda**

Thailand

**Victor H Aquino**

Brazil

**Juan Arbiza**

Uruguay

**Carlos Arias**

Mexico

**Jiro Arikawa**

Japan

**Minetaro Arita**

Japan

**Luciana Arruda**

Brazil

**Jose T. Ascencio-Ibanez**

USA

**Antonio Ascione**

Italy

**Sassan Asgari**

Australia

**Claire Atkinson**

UK

**Eduardo Azziz-Baumgartner**

USA

**Leen Baert**

Switzerland

**Olfa Bahri**

Tunisia

**Susan Baker**

USA

**Judith Ball**

USA

**Subrata Banerjee**

India

**Celia Barardi**

Brazil

**Edel Barbosa-Stancioli**

Brazil

**Michael Baron**

UK

**John Barr**

UK

**Erik Barton**

USA

**Chris Basler**

USA

**Lorena Bavia**

Brazil

**Bhupinder Bawa**

USA

**Eduardo Bejarano**

Spain

**Brendan Bell**

Canada

**Dirk Bellstedt**

South Africa

**Jessica Belser**

USA

**Dennis Bente**

USA

**Vincent Beringue**

France

**Ben Berkhout**

Netherlands

**Jesus F Bermejo-Martin**

Spain

**Charles Berry**

USA

**Mael Bessaud**

France

**Ramya Bhatia**

UK

**Bishnupriya Bhattacharya**

UK

**Huijie Bian**

China

**Marina Bidin**

Croatia

**John Blaho**

USA

**Sandra Blome**

Germany

**Hubert Blum**

Germany

**Karl Boehme**

USA

**Andrew Bohm**

USA

**Myrna Bonaldo**

Brazil

**Cláudio Bonjardim**

Brazil

**Alessandra Borges**

Brazil

**Guglielmo Borgia**

Italy

**Silvia Boscardin**

Brazil

**Crystal Boudreaux**

USA

**Andrew Bowman**

USA

**Paulo Brandao**

Brazil

**Laurence Briant**

France

**David Brighty**

UK

**Christopher Broder**

USA

**Ivan Brukner**

Canada

**Hubert Buczkowski**

UK

**Gertrude Buehring**

USA

**Alexander Bukreyev**

USA

**Cara Burns**

USA

**Michael Burwinkel**

Germany

**Pierre Busson**

France

**James Campbell**

Canada

**Patricia Cane**

UK

**Chunxia Cao**

USA

**Ya Cao**

China

**Antonella Caputo**

Italy

**Tereza Cardoso**

Brazil

**Faizal Careem**

Canada

**Claudio Caruso**

Italy

**Elena Catelli**

Italy

**Luciano Cavalcanti**

Brazil

**Mattia Cecchinato**

Italy

**Remi Charrel**

France

**Animesh Chatterjee**

USA

**Yahia Chebloune**

France

**Gerald Kimani Chege**

South Africa

**En-Qiang Chen**

China

**Jen-Yang Chen**

Taiwan

**Jianfei Chen**

China

**Ji-Ming Chen**

China

**Limin Chen**

Canada

**Quanjiao Chen**

China

**Tong-Xin Chen**

China

**Xinwen Chen**

China

**Zhongbin Chen**

China

**Anchun Cheng**

China

**Tong Cheng**

China

**Zhaobang Cheng**

China

**Sarah Cherian**

India

**Bor-Luen Chiang**

Taiwan

**Chiara Chiapponi**

Italy

**Mohamad Chikh Ali**

USA

**V Gregory Chinchar**

USA

**Hui-Ling Chiou**

Taiwan

**Christopher Chiu**

UK

**Susan Chiu**

Hong Kong

**Kyoung-Oh Cho**

Korea, South

**Bum Soon Choi**

Korea, South

**Jin Huk Choi**

USA

**Kyoung-Seong Choi**

Korea, South

**Pele Chong**

Taiwan

**Yen-Hung Chow**

Taiwan

**Wan-Long Chuang**

Taiwan

**Pauline Chugh**

USA

**Konstantin Chumakov**

USA

**Ju-Young Chung**

Korea, South

**Massimo Ciccozzi**

Italy

**Christine Elisabeth Clavel**

France

**Carla Coffin**

Canada

**Philippe Colson**

France

**Sarah Connolly**

USA

**Samuel Cordey**

Switzerland

**Anthony Corfield**

UK

**Alexandre Costa**

Brazil

**Andre Costa-da-Silva**

Brazil

**Maria Isabel Costafreda**

USA

**Veronica Costantini**

USA

**Therese Couderc**

Gabon

**Brenda Coutts**

Australia

**Manuel Crespo**

Spain

**Helen Crooke**

UK

**Robert Cross**

USA

**Edson da Silva**

Brazil

**Ajmal Dalwai**

Kuwait

**Armando Damiani**

Germany

**Elsa Damonte**

Argentina

**Andrew Davidson**

UK

**Mohamed Ali Daw**

Libya

**Craig Day**

USA

**Damián F. de Andrés**

Spain

**Luana de Borba**

Argentina

**Juan Carlos de la Torre**

USA

**Carlos de Noronha**

USA

**Robert de Vries**

USA

**Emmie de Wit**

USA

**Nicola Decaro**

Italy

**Rosa del Angel**

Mexico

**Jose Del Campo**

Spain

**Adriana Delfraro**

Uruguay

**Gustavo Delhon**

USA

**Eric Delwart**

USA

**Joachim Denner**

Germany

**Moutaz Derbala**

Qatar

**Tararaj Dharakul**

Thailand

**Adarsh Dharan**

USA

**Barbara Di Martino**

Italy

**Detlef E. Dietrich**

Germany

**Paul Digard**

UK

**Chan Ding**

China

**Ulf Dittmer**

Germany

**Maurizio Divizia**

Italy

**Stephen Dollery**

USA

**Irini Doytchinova**

Bulgaria

**Jan Felix Drexler**

Germany

**Edward Dubovi**

USA

**Robert Duda**

USA

**Stephen Dunham**

UK

**Rebecca Dutch**

USA

**Andrew Easton**

UK

**Jose Echevarria**

Spain

**Maryna Eichelberger**

USA

**Anna Eis-Huebinger**

Germany

**Manal El-Sayed**

Egypt

**Othmar Engelhardt**

UK

**Luis Enjuanes**

Spain

**Jose M Escribano**

Spain

**Pedro Esteves**

Portugal

**Jennifer Evans**

UK

**James Evermann**

USA

**Catherine Ewen**

USA

**Shufang Fan**

USA

**Minggang Fang**

USA

**A. Hossain Farid**

Canada

**Francesco Feletti**

Italy

**Wenhai Feng**

China

**Ricardo Flores**

Spain

**Robert Flower**

Australia

**Caroline Fossum**

Sweden

**Jeffrey Foster**

USA

**Sandra Frabasile**

Uruguay

**Rennos Fragkoudis**

UK

**Silvia Franceschi**

France

**Conrad Freuling**

Germany

**Jean-Pierre Frossard**

UK

**Cristina Fuentes Pardo**

Spain

**Masashi Fukayama**

Japan

**Monica Galiano**

UK

**Donato Gallitelli**

Italy

**Feng Gao**

China

**Feng Gao**

USA

**Antonio Garmendia**

USA

**Lana Garmire**

USA

**Silvana Gaudieri**

Australia

**Ritu Gaur**

India

**Brian Geiss**

USA

**Elke Genersch**

Germany

**Abdeljelil Ghram**

Tunisia

**Adrian Gibbs**

Australia

**Ivone Giffard-Mena**

Mexico

**Jason Gill**

USA

**Andre Gomes**

Brazil

**Jordi Gomez**

Spain

**Esperanza Gomez-Lucia**

Spain

**Jean-Paul Gonzalez**

USA

**Radhika Gopal**

USA

**Hideo Goto**

Japan

**Maruthi M N Gowda**

UK

**Urs Greber**

Switzerland

**Giorgio Gribaudo**

Italy

**Anthony Griffiths**

USA

**Charles Grose**

USA

**Christian Grund**

Germany

**Linlin Gu**

USA

**Rodrigo Guabiraba**

France

**Larisa Gubareva**

USA

**Maria Isabel Guedes**

Brazil

**Hailong Guo**

USA

**Haitao Guo**

USA

**Xiaofeng Guo**

China

**Ahmet Gurakar**

USA

**Jon Gutierrez**

Slovenia

**Clifford Guy**

USA

**James Guy**

USA

**Bart Haagmans**

Netherlands

**Hans Haecker**

USA

**Roy Hall**

Australia

**Xinbing Han**

USA

**Ken-Ichi Hanaki**

Japan

**Tomas Hanke**

UK

**Kathryn Hanley**

USA

**Chetan Hans**

USA

**Gordon Harkins**

South Africa

**Heli Harvala**

UK

**Graham Hatfull**

USA

**Daisuke Hayasaka**

Japan

**Qigai He**

China

**Isabelle Heard**

France

**Alireza Heidari**

Italy

**Terho Heikkinen**

Finland

**Regine Heilbronn**

Germany

**Andrew Henderson**

USA

**Lara Herrero**

Australia

**Guy Hervé**

France

**Toshiki Himeda**

Japan

**Jung Joo Hong**

USA

**Daniel Horton**

UK

**Katja Hoschler**

UK

**Margaret Hosie**

UK

**Hak Hotta**

Japan

**Weihong Hou**

USA

**Cheng Huang**

USA

**Li-Rung Huang**

Taiwan

**Yaowei Huang**

USA

**Yu Huang**

China

**Christine Hughes**

Canada

**Ivan Hung**

Hong Kong

**Elizabeth Hunsperger**

Puerto Rico

**Dae Young Hur**

Korea, South

**Aeron Hurt**

Australia

**Gisela Hussey**

USA

**Edward Hutchinson**

UK

**Ali Idris**

USA

**Kyung-Soo Inn**

Korea, South

**Will Irving**

UK

**Charles Isaacs**

USA

**Masashi Iwanaga**

Japan

**Alan Jackson**

Canada

**Richard Jackson**

UK

**Daral Jackwood**

USA

**Lisa Jameson**

UK

**Shijin Jiang**

China

**Emma Job**

Australia

**Reimar Johne**

Germany

**Nicholas Johnson**

UK

**Ho Won Jung**

Korea, South

**Sandra Junglen**

Germany

**Adrian Kajon**

USA

**Robert Kalejta**

USA

**Tatsuo Kanda**

USA

**Hisatoshi Kaneko**

Japan

**Amit Kapoor**

USA

**Hiroaki Kariwa**

Japan

**Dietmar Katinger**

Austria

**Benedikt Kaufer**

Germany

**Ghazi Kayali**

USA

**Alyson Kelvin**

Canada

**Pattara Khamrin**

Thailand

**Mohsin Khan**

USA

**Madhu Khanna**

India

**Sunil Khattar**

USA

**Alexander Khromykh**

Australia

**Hyun Soo Kim**

Korea, South

**Seong-Jun Kim**

USA

**Shin-Hee Kim**

USA

**David Kimberlin**

USA

**Lóránt Király**

Hungary

**Peter Kirkland**

Australia

**Irina Kiseleva**

Russia

**Leera Kittigul**

Thailand

**Boris Klempa**

Slovakia

**Maja Kodani**

USA

**Jeffrey Koehler**

USA

**Andrea Koishi**

Brazil

**Eva Krause**

Germany

**Astrid Krmpotic**

Croatia

**Andrew Kropinski**

Canada

**Peter Krug**

USA

**Detlev H Kruger**

Germany

**Claude Krummenacher**

USA

**Diogo Kuczera**

Brazil

**Ajit Kumar**

USA

**Sachin Kumar**

India

**Stefan Kunz**

Switzerland

**Lise Kirstine Kvisgaard**

Denmark

**Giuseppina La Rosa**

Italy

**Ole Lagatie**

Belgium

**Alexander Lai**

Hong Kong

**Yinghua Lan**

China

**Stephen E. Lapinsky**

Canada

**Tomasz Laskus**

Poland

**Susanna Lau**

Hong Kong

**Karen Laurie**

Australia

**James Lawson**

Australia

**Jen Layden**

USA

**Thomas Layden**

USA

**Chuan-Mo Lee**

Taiwan

**Joong Bok Lee**

Korea, South

**David Lefebvre**

Belgium

**Elliot Lefkowitz**

USA

**Guy Lemay**

Canada

**Niels A.W. Lemmermann**

Germany

**Min Levine**

USA

**Avram Levy**

Australia

**Jay Levy**

USA

**Chengjun Li**

China

**Lanjuan Li**

China

**Linlin Li**

USA

**Mei-Ling Li**

USA

**Shifang Li**

China

**Min Liao**

China

**Daniel Libraty**

USA

**Magnus Lindh**

Sweden

**Fenyong Liu**

USA

**Jinhua Liu**

China

**Qinfang Liu**

USA

**Francisco Lobo**

Brazil

**Martin Loessner**

Switzerland

**Huaguang Lu**

USA

**Michaela Lucas**

Australia

**Juan Ludert**

Mexico

**Catherine Luke**

USA

**Shuhong Luo**

China

**Blanca Lupiani**

USA

**Hinh Ly**

USA

**Samantha Lycett**

UK

**Daoxin Ma**

China

**Guanggang Ma**

China

**Liqing Ma**

China

**Yijie Ma**

USA

**Dominiek Maes**

Belgium

**Padmanabhan Mahadevan**

USA

**Eugene Major**

USA

**Shinji Makino**

USA

**Carlos Maluquer de Motes**

UK

**Nicholas Maness**

USA

**Balaji Manicassamy**

USA

**Anna Liza Manlutac**

USA

**Spilios Manolakopoulos**

Greece

**Atika Mansoor**

Pakistan

**María Rosa Marano**

Argentina

**Sylvie Marché**

Belgium

**Denise Marston**

UK

**Contigiani Marta**

Argentina

**Darren Martin**

South Africa

**Miguel A Martin-acebes**

Spain

**Isidoro Martinez**

Spain

**Miguel Angel Martinez**

Spain

**Encarna Martinez Salas**

Spain

**Javier Martinez-Picado**

Spain

**Luis Martinez-Sobrido**

USA

**Regina Martins**

Brazil

**Vilma Martins**

Brazil

**William Mason**

USA

**Enric Mateu**

Spain

**Masanori Matsui**

Japan

**Yumiko Matsuoka**

USA

**Shohei Matsuura**

Japan

**Giada Mattiuzzo**

UK

**Leena Maunula**

Finland

**Sebastian Maurer-Stroh**

Singapore

**Axel Mauroy**

Belgium

**Budhaditya Mazumdar**

USA

**Urszula Mazurek**

Poland

**Julie McAuley**

Australia

**Harris McFerrin**

USA

**Gerald McInerney**

Sweden

**John McKillen**

UK

**Gary McLean**

UK

**José A. Melero**

Spain

**Baozhong Meng**

Canada

**Janet Mertz**

USA

**Andrew Mesecar**

USA

**Francois Meurens**

Canada

**Florencia Meyer**

Uruguay

**Andreas Meyerhans**

Spain

**Scott Michael**

USA

**Thomas Michalak**

Canada

**Luiz Miletti**

Brazil

**David Milich**

USA

**Patti Miller**

USA

**Hyeyoung Min**

Korea, South

**Chad Mire**

USA

**Nischay Mishra**

USA

**Tadayoshi Mitsuhashi**

Japan

**Katsumi Mizuta**

Japan

**Susanne Modrow**

Germany

**Ugo Moens**

Norway

**Ka Pun Mok**

Hong Kong

**Eamonn Molloy**

Ireland

**Isabella Monne**

Italy

**Ronald Montelaro**

USA

**Emanuele Montomoli**

Italy

**Cary Moody**

USA

**Christiane Moog**

France

**Martin Moore**

USA

**Tsafrir Mor**

USA

**Luiz Tadeu Moraes Figueiredo**

Brazil

**Norio Mori**

Japan

**Enrique Moriones**

Spain

**Rhoda Morrow**

USA

**Eric Mossel**

USA

**Bruno Mota**

Brazil

**Nissin Moussatche**

USA

**Sunil Mukherjee**

India

**Marcel Müller**

Germany

**Patricia Mulrooney-Cousins**

Canada

**Muhammad Munir**

UK

**Yoshiki Murakami**

Japan

**William Murphy**

USA

**Jonah Musa**

Nigeria

**Yael Mutsafi**

Israel

**Greg Mutze**

Australia

**Eva Nagy**

Canada

**Keisuke Nakagawa**

Japan

**Scott Napper**

Canada

**Krishna Narayanan**

USA

**Farooq Nasar**

USA

**Beatriz Navarro**

Italy

**Jesus Navas-Castillo**

Spain

**Somanna Naveen**

USA

**Clive Naylor**

UK

**Jean Ndjomou**

USA

**Daniel Nelson**

USA

**Laura Newcomb**

USA

**Johan Neyts**

Belgium

**Terry Fei Fan Ng**

USA

**Tsai Nga Wing Polly**

Hong Kong

**John Nicholls**

Hong Kong

**Zuoming Nie**

China

**Veljko Nikolin**

Germany

**Yorihiro Nishimura**

Japan

**Andreas Nitsche**

Germany

**Juliana Nogueira**

Brazil

**Helene Norder**

Sweden

**Marcio Nunes**

Brazil

**Mark O'Dea**

Australia

**Leslie Ogorzaly**

Luxembourg

**Aileen O'Hearn**

USA

**Seii Ohka**

Japan

**Judith Olejnik**

USA

**Gene Olinger**

USA

**Hugo Oliveira**

Portugal

**John Daniel O'Neil**

UK

**Eleonore Ostermann**

Germany

**Masao Ota**

Japan

**David Ott**

USA

**Qishui Ou**

China

**Paula Padula**

Argentina

**Anandan Paldurai**

USA

**Peter Palese**

USA

**Ann Palmenberg**

USA

**Ana Luiza Pamplona Mosimann**

Brazil

**Mary Pantin-Jackwood**

USA

**Hanu Pappu**

USA

**Mark Parcells**

USA

**Satya Parida**

UK

**Joanna Parish**

UK

**Bongkyun Park**

Korea, South

**Rebecca Parr**

USA

**Colin Parrish**

USA

**Kunj Pathak**

USA

**John Patton**

USA

**Ben Peeters**

Netherlands

**Daxin Peng**

China

**Yihong Peng**

China

**Luciane Maria Perazzolo**

Brazil

**Carlos Pereira**

Brazil

**Jean-Pierre Perreault**

Canada

**Martin Pfeffer**

Germany

**Sébastien Pfeffer**

France

**Tram Pham**

Canada

**Tung Phan**

USA

**Nittaya Phanuphak**

Thailand

**Jaime Pignatelli**

Spain

**Gorben Pijlman**

Netherlands

**Benjamin Pinsky**

USA

**Aguinaldo Pinto**

Brazil

**Rosa Maria Pintó**

Spain

**Antonio Piralla**

Italy

**Mauro Pistello**

Italy

**Justin Pita**

USA

**Stefan Pöhlmann**

Germany

**Welkin Pope**

USA

**Hamid Pourianfar**

Iran

**Ali Pouryasin**

Iran

**Ramesh Prabhu**

USA

**Martina Prelog**

Germany

**Joseph Prescott**

USA

**Cinta Prieto**

Spain

**Krzysztof Pyrc**

Poland

**Zhikang Qian**

China

**Chao Qiu**

China

**Jianming Qiu**

USA

**Josep Quer**

Spain

**Shafquat Rafiq**

UK

**Jaya Rajaiya**

USA

**Yogendra Rajawat**

USA

**Huiying Rao**

China

**Adam Rash**

UK

**Thomas Bruun Rasmussen**

Denmark

**Jorgen Ravoet**

Belgium

**William Rawlinson**

Australia

**Simon Rayner**

China

**Cath Rees**

UK

**Ilona Reimann**

Germany

**Chantal Reusken**

Netherlands

**Mathilde Richard**

Netherlands

**Elizabeth Rieder**

USA

**Daniel Ripoll**

USA

**Guillermo Risatti**

USA

**Pierre Rivailler**

USA

**Rafael F. Rivera-Bustamante**

Mexico

**Steve Rockman**

Australia

**Barry Rockx**

USA

**Julio Rodriguez**

Australia

**Pierre Roques**

France

**Daniela Rosa**

Brazil

**German Rosas-Acosta**

USA

**Anca Roseanu**

Romania

**Helene Rosenberg**

USA

**Susan Ross**

USA

**Michael G Rossmann**

USA

**Raymond Rowland**

USA

**Dora Ruchansky**

Uruguay

**Franco Maria Ruggeri**

Italy

**Nicolas Ruggli**

Switzerland

**Alvaro Ruiz**

Chile

**Jonathan Runstadler**

USA

**Wi-Sun Ryu**

Korea, South

**Armin Saalmueller**

Austria

**Ali Sabahi**

USA

**Ivan Sadowski**

Canada

**Mahmut Safak**

USA

**Dipongkor Saha**

USA

**Nitin Saksena**

Australia

**Francisco Javier Salguero**

UK

**Teruo Sano**

Japan

**Flavia Santos**

Brazil

**Daniel Santos Mansur**

Brazil

**Yoshikuni Sato**

Japan

**Benjamin Satterfield**

USA

**Andreas Sauerbrei**

Germany

**Carita Savolainen-Kopra**

Finland

**Hirofumi Sawa**

Japan

**Paula Schaffer**

USA

**Luis Schang**

Canada

**Horst Schirrmeier**

Germany

**Jeffrey Schneider**

USA

**Juergen Schneider-Schaulies**

Germany

**Amy Schuh**

USA

**Anna Charlotte Schultz**

Denmark

**Martin Schwemmle**

Germany

**Michel Segondy**

France

**Bert Semler**

USA

**Noemi Sevilla**

Spain

**Norazizah Shafee**

Malaysia

**Ruiping She**

China

**Noula Shembade**

USA

**Huigang Shen**

China

**Tao Shen**

China

**Jishu Shi**

USA

**Yusuke Shimakawa**

Japan

**Maya Shmulevitz**

Canada

**Guilherme Silveira**

Brazil

**Rodrigo Silvestre**

Brazil

**Valerie Sim**

Canada

**Peter Simmonds**

UK

**David Skibinski**

Singapore

**Mikael Skurnik**

Finland

**Anny Slama-Schwok**

France

**Marek Smieja**

Canada

**Teemu Smura**

Finland

**Chantal Snoeck**

Luxembourg

**Muhammad Sohail**

USA

**Chang-Seon Song**

Korea, South

**Daniele Souza**

Brazil

**Doris Souza**

Brazil

**Erica Spackman**

USA

**Tim Sparer**

USA

**Fernando Spilki**

Brazil

**Burke Squires**

USA

**Peter Staeheli**

Germany

**Thomas Stamminger**

Germany

**Martin Steinau**

USA

**Bettie Steinberg**

USA

**Stephen Stray**

USA

**Rebecca Strong**

UK

**Daisy Strottmann**

Brazil

**Elankumaran Subbiah**

USA

**Christopher Sullivan**

USA

**Jack M. Sullivan**

USA

**Artur Summerfield**

Switzerland

**Rebecca Sumner**

UK

**Petri Susi**

Finland

**Leonardo Susta**

USA

**Gerd Sutter**

Germany

**Troy Sutton**

USA

**Gulam Syed**

USA

**Yoichi Takahagi**

Japan

**Akihiro Tamori**

Japan

**Yee-Joo Tan**

Singapore

**Yasuhito Tanaka**

Japan

**Xianchun Tang**

USA

**Yinghua Tang**

China

**Yi-Wei Tang**

USA

**Xiaorong Tao**

China

**Vera Tarakanova**

USA

**Bart Tarbet**

USA

**Paul Targett-Adams**

Sweden

**Kaspars Tars**

Latvia

**Adam Taylor**

Australia

**John Taylor**

USA

**Maureen Taylor**

South Africa

**Paul Thomas**

USA

**Peter Tijssen**

Canada

**Andrey Tokarev**

USA

**Thomas Tran**

Australia

**Ramona Trebbien**

Denmark

**Alfonsina Trento**

Spain

**Stephen Kwok-Wing Tsui**

Hong Kong

**Kenji Tsukamoto**

Japan

**Changchun Tu**

China

**Edan Tulman**

USA

**Tobias Tuthill**

UK

**Sukathida Ubol**

Thailand

**Takeji Umemura**

Japan

**Wim Van der Poel**

Netherlands

**James Van Etten**

USA

**Frank van Kuppeveld**

Netherlands

**Herve Vanderschuren**

Switzerland

**Arvind Varsani**

New Zealand

**Francisco Veas**

France

**Michael Veit**

Germany

**Georges Verjans**

Netherlands

**Newton Medeiros Vidal**

Brazil

**Alexandre Vieira Machado**

Brazil

**Dhanasekaran Vijaykrishna**

Singapore

**Stefan Vilcek**

Slovakia

**Heiner von Buttlar**

Germany

**Ann Vossen**

Netherlands

**Abdul Waheed**

USA

**Peter Walker**

Australia

**Per Wallgren**

Sweden

**Hongquan Wan**

USA

**Xiu-Feng (Henry) Wan**

USA

**Jianwei Wang**

China

**Robert YL Wang**

Taiwan

**Tian Wang**

USA

**Xiaomei Wang**

China

**Wanzhe Yuan**

China

**Gulam Waris**

USA

**Peter Wark**

Australia

**Simone Warner**

Australia

**Shumpei Watanabe**

Japan

**Koichi Watashi**

Japan

**Nadia Wauquier**

France

**Eefke Weesendorp**

Netherlands

**Lai Wei**

China

**Manfred Weidmann**

UK

**Jonas Wensman**

Sweden

**Judith White**

USA

**Gary Whittaker**

USA

**Chris Wiethoff**

USA

**Rebecca Wilkes**

USA

**David Williams**

Australia

**William Wilson**

USA

**Helen Wise**

UK

**Roland Wolkowicz**

USA

**Sek-Man Wong**

Singapore

**Gary Wormser**

USA

**Grzegorz Jan Wozniakowski**

Poland

**Changde Wu**

China

**Ho-sheng Wu**

Taiwan

**Dongbo Wu**

China

**Xiaofeng Wu**

China

**Zhiyong Xi**

USA

**Shi-Hua Xiang**

USA

**Shu-Yuan Xiao**

USA

**Peng Xie**

China

**Youhua Xie**

China

**Xiyan Xu**

USA

**Kentaro Yamada**

Japan

**Seiya Yamayoshi**

Japan

**Hanchun Yang**

China

**Hwai-I Yang**

Taiwan

**Kui Yang**

USA

**Elizabeth Yanik**

USA

**Yoshihiko Yano**

Japan

**Chau-Ting Yeh**

Taiwan

**Man Lung Yeung**

Hong Kong

**Shounan Yi**

Australia

**Lawrence Young**

UK

**Youxiang Diao**

China

**Di Yu**

Australia

**Li Yu**

China

**Ming-Lung Yu**

Taiwan

**Qingzhong Yu**

USA

**Hang Yuan**

USA

**Yuchen Fan**

China

**Zeng Yue**

China

**Yuehuan Liu**

China

**Kwok Yung Yuen**

Hong Kong

**Nadezhda Yun**

USA

**Syed Sohail Zahoor Zaidi**

Pakistan

**Juan Zapata**

USA

**Hassan Zaraket**

Lebanon

**Mirjam Zeisel**

France

**Bo Zhang**

China

**Dabing Zhang**

China

**Gui-Hong Zhang**

China

**Hengmu Zhang**

China

**Hong Zhang**

USA

**Jinyang Zhang**

China

**Ping Zhang**

China

**Jincun Zhao**

USA

**Jinzhuo Zhao**

China

**Weihao Zheng**

China

**Zhenyu Zhong**

USA

**Bin Zhou**

USA

**En-Min Zhou**

China

**Fengfeng Zhou**

China

**Guohui Zhou**

China

**Jiyong Zhou**

China

**Hua Zhu**

USA

